# Relationship of imatinib-free plasma levels and target genotype with efficacy and tolerability

**DOI:** 10.1038/sj.bjc.6604355

**Published:** 2008-05-06

**Authors:** N Widmer, L A Decosterd, S Leyvraz, M A Duchosal, A Rosselet, M Debiec-Rychter, C Csajka, J Biollaz, T Buclin

**Affiliations:** 1 Division of Clinical Pharmacology, Department of Medicine, Centre Hospitalier Universitaire Vaudois and University of Lausanne, Lausanne, Switzerland; 2 Multidisciplinary Oncology Centre, Centre Hospitalier Universitaire Vaudois and University of Lausanne, Lausanne, Switzerland; 3 Service of Haematology, Department of Medicine, Centre Hospitalier Universitaire Vaudois and University of Lausanne, Lausanne, Switzerland; 4 Department of Human Genetics, University of Leuven and University Hospital Gasthuisberg, Leuven, Belgium

**Keywords:** imatinib, pharmacokinetics and pharmacodynamics, pharmacogenetics, leukaemia, sarcoma, signal transduction inhibitors

## Abstract

Imatinib has revolutionised the treatment of chronic myeloid leukaemia (CML) and gastrointestinal stromal tumours (GIST). Using a nonlinear mixed effects population model, individual estimates of pharmacokinetic parameters were derived and used to estimate imatinib exposure (area under the curve, AUC) in 58 patients. Plasma-free concentration was deduced from a model incorporating plasma levels of alpha_1_-acid glycoprotein. Associations between AUC (or clearance) and response or incidence of side effects were explored by logistic regression analysis. Influence of *KIT* genotype was also assessed in GIST patients. Both total (in GIST) and free drug exposure (in CML and GIST) correlated with the occurrence and number of side effects (e.g. odds ratio 2.7±0.6 for a two-fold free AUC increase in GIST; *P*<0.001). Higher free AUC also predicted a higher probability of therapeutic response in GIST (odds ratio 2.6±1.1; *P*=0.026) when taking into account tumour *KIT* genotype (strongest association in patients harbouring exon 9 mutation or wild-type *KIT*, known to decrease tumour sensitivity towards imatinib). In CML, no straightforward concentration–response relationships were obtained. Our findings represent additional arguments to further evaluate the usefulness of individualising imatinib prescription based on a therapeutic drug monitoring programme, possibly associated with target genotype profiling of patients.

Imatinib mesylate (Glivec®; Novartis, Basel, Switzerland) has revolutionised the treatment and prognosis of chronic myeloid leukaemia (CML) (Druker, 2003; Tothova *et al*, 2005) and gastrointestinal stromal tumours (GIST) (Steinert *et al*, 2005). Imatinib was rationally designed to inhibit the PDGF receptor and the BCR-ABL tyrosine kinase (the hallmark of CML), and it was also found to potently inhibit autophosphorylation of the tyrosine kinase receptor c-KIT (involved in the pathogenesis of GIST) (Demetri *et al*, 2002).

BCR-ABL kinase results from a reciprocal t(9,22) translocation that gives rise to the Philadelphia chromosome in CML (Capdeville *et al*, 2002). Constitutive activation of c-KIT, associated with various mutation profiles, is observed in the majority of GISTs. The most common mutation site of *KIT* is located on exon 11. Exon 9 mutation occurs in 10–15% of patients, defining a distinct subset of GISTs having an aggressive clinical behaviour. A few GISTs are characterised by another mutations profile, and about 10% of patients have undetectable mutations (wild type, wt) (Antonescu *et al*, 2005).

Treatment with tyrosine kinase inhibitors such as imatinib is considered at present to be taken indefinitely, owing to the apparent insensitivity of stem cells to imatinib (Michor *et al*, 2005; Michor, 2007). Moreover, they are not devoid of inconvenience and toxicity, and resistance occurs in a significant number of patients (Weisberg and Griffin, 2003; Hochhaus and La Rosee, 2004). Finally, such therapies remain fairly expensive at this time (Simonsson *et al*, 2007). Various adverse events have been described for imatinib, including fluid retention, nausea, skin rash and muscle cramps, with an incidence of more than 50% (grades 1–4) (Cohen *et al*, 2005; Zalcberg *et al*, 2005). Cardiotoxicity has also been recently reported (Kerkela *et al*, 2006). Cellular mechanisms of resistance in CML include point mutations in *BCR-ABL* gene (up to 40 identified), *BCR-ABL* amplification or activation of alternative survival signalling pathways (Sawyers *et al*, 2002; Weisberg and Griffin, 2003). For GISTs, the tumour genotype is a predictor of response to imatinib. Patients harbouring tumours characterised by an exon 11 *KIT* mutation may benefit from a better response to imatinib compared to other subgroups, notably exon 9 mutants or wt *KIT* tumours (Heinrich *et al*, 2003; Debiec-Rychter *et al*, 2006). Molecular analysis of GISTs thus appears to be an important clinical tool to identify patients at high risk of disease progression. Moreover, about half of the imatinib-resistant GIST patients had acquired secondary mutations in the kinase domain of c-KIT (Antonescu *et al*, 2005).

Additionally, resistance could also be directly or indirectly caused by an increase in cellular efflux of imatinib, mediated mainly by the drug transporter P-gp (P-glycoprotein) (Mahon *et al*, 2003; Widmer *et al*, 2007), or by a decrease in cellular influx, mediated by the uptake carrier hOCT1 (organic cation transporter) (Thomas *et al*, 2004; Crossman *et al*, 2005; Wang *et al*, 2008). Host-dependent mechanisms of resistance have also been incriminated, including modulation of imatinib binding to *α*_1_-acid glycoprotein (AGP) in plasma (Gambacorti-Passerini *et al*, 2000; Gambacorti-Passerini *et al*, 2003; Larghero *et al*, 2003) and/or possibly enhanced drug metabolism (Rochat *et al*, 2008). Finally, nonadherence to imatinib dosage regimen may also play a role in resistance (Tsang *et al*, 2006). A given dose therefore yields very different circulating concentrations between patients (Widmer *et al*, 2006; Larson *et al*, 2008), possibly favouring the selection of resistant cellular clones in case of subtherapeutic drug exposure.

Several pharmacokinetic (PK) studies have been carried out for imatinib. Some have been able to verify the influence of factors such as weight, albuminaemia, haemoglobinaemia or *ABCB1* (*MDR1*) polymorphism on its PK (Judson *et al*, 2005; Schmidli *et al*, 2005; Gurney *et al*, 2007) but not of those such as hepatic enzymes or impaired liver or kidney function (Widmer *et al*, 2006; Gibbons *et al*, 2008; Ramanathan *et al*, 2008). Furthermore, recent evidence suggests that steady-state trough imatinib plasma concentration (TPC) at initiation of therapy is a significant predictor of complete cytogenetic and major molecular responses (Larson *et al*, 2008). TPC also appears to correlate with response in CML (Picard *et al*, 2007) as well as in GIST (Demetri *et al*, 2008). Interestingly, recent studies have begun to investigate the free drug exposure of imatinib (Delbaldo *et al*, 2006; Widmer *et al*, 2006). The study from Delbado also explored the relationship between drug exposure (area under the curve, AUC) and effect. It showed that unbound drug exposure was correlated to the haematological toxicity (absolute neutrophil count), but it did not find significant association with treatment efficacy in GIST patients. However, the modulating influence of tumour genetics on the concentration–effect relationship of imatinib, and similar new targeted anticancer drugs, certainly deserves additional evaluation.

The aims of this clinical investigation were as follows: (1) to explore further PK–PD relationships in a population of CML and GIST patients, and (2) to evaluate the specific influence of the target genotype on this relationship in the GIST sub-population.

## MATERIALS AND METHODS

### Study population and genetic characterisation

The present PK–PD (pharmacokinetic–pharmacodynamic) analysis was performed using data from 58 patients, out of 59 who provided plasma samples collected over 3 years (Widmer *et al*, 2006). For the present analysis, 280 plasma samples were considered (corresponding to routine visits only). This observational study was approved by the Ethics Committee of the Lausanne Faculty of Medicine. Informed written consent was obtained from all the participants.

The population PK analysis of these data has been published elsewhere (Widmer *et al*, 2006). The patients included in the present analysis were 38 with GIST and 20 with CML, who received imatinib at various dosage regimens (150–800 mg daily). Peripheral blood samples, obtained under steady-state conditions, were drawn periodically at 1- to 6-month intervals on follow-up visits, along with routine laboratory tests. In addition to accurate dosing and sampling time information, a comprehensive set of demographic and biological data were recorded for each patient, including plasma AGP (Widmer *et al*, 2006).

Imatinib concentration was measured using a validated method by high-performance liquid chromatography after solid phase extraction (Widmer *et al*, 2004). The lower limit of quantification is 50 *μ*g l^−1^, the mean interday coefficient of variation is lower than 2.4% and the range of interday deviations is within −0.6 to +0.7%.

The tumour genetic profile of 20 GIST patients was assessed at the time of the multicentric EORTC Soft Tissue and Bone Sarcoma trial (Debiec-Rychter *et al*, 2006). Genomic DNA was extracted from sections of paraffin-embedded tumour blocks. Exons 9, 11, 13 and 17 of the *KIT* gene were amplified by PCR, and the amplicons were analysed for mutations by a combination of DHPLC pre-screening (WAVE DHPLC system, Transgenomic, Cramlington, UK) and bidirectional sequencing (Debiec-Rychter *et al*, 2004). Specimens that had no detectable *KIT* mutation (wt *KIT*) were further tested for *PDGFRA* exons 12 and 18 mutations. The genetic profiles were coded on a binary scale, with 1=presence of mutation known to confer resistance to imatinib treatment (mutation on *KIT* exon 9 or wt profile) and 0=absence of such mutation (*KIT* exon 11 mutation).

### Assessment of imatinib exposure

On the basis of model purposely developed at the time of our population PK study (nonlinear mixed effects model; NONMEM) (Widmer *et al*, 2006), individual *post hoc* Bayesian estimates of PK parameters were derived for all samples. They were used to calculate maximum likelihood individual drug exposure levels, expressed as AUC (defined as Dose/CL·*τ*, where CL is the clearance and *τ* the dosing interval).

Moreover, free parameters (i.e. corresponding to the unbound drug) were estimated using the PK model incorporating plasma AGP levels that we formerly developed (Widmer *et al*, 2006).

### Assessment of clinical response

The therapeutic response was determined at the time of routine follow-up visits. For CML, it was coded on a 3-point scale (complete, CHR=2, partial, PHR=1 and absent, NHR=0 haematological remission, based on white-blood cell count), and was in accordance with RECIST criteria for GIST (Therasse *et al*, 2000). This criterion was recoded at the time of the efficacy analysis into a 2-point scale (overall responses (OR=1), comprising complete response (CR) plus partial response (PR) *vs* stable disease (SD) plus progressive disease=0).

As standardised evaluation of typical side effects was not systematically available in our patient's population (e.g. National Cancer Institute's Common Toxicity Criteria, NCI-CTC), the number of side effects experienced by patients was considered instead as a surrogate outcome for toxicity (summarised in a 4-point scale; 0, 1, 2 and 3 or more side effects).

For each blood sample collected, the efficacy and toxicity scores, as well as the Dose considered, were the ones corresponding or reported at the time of sampling. Every score was double-checked before PK–PD analysis.

### Statistics

A concentration–effect exploration was first carried out in CML and GIST patients. Associations between log-transformed Dose, as well as total and free AUC or CL, and therapeutic response or toxicity, were explored by ordered logistic regression analysis (Stata® version 8.2, Stata Co., College Station, TX, USA) (Stata Corp, 2003). Although this per-sample analysis allowed taking into account the variations along the time of dose, AGP levels, body weight and age, a more stringent per-patient analysis was also performed to keep away from intrapatient correlation issues. To that purpose, all different data were collapsed in one value for each patient (i.e. average Dose, AUC and CL *vs* median efficacy and toxicity scores).

In the GIST sub-population, the influence of target mutation profile on the therapeutic response was additionally assessed by incorporating the patients' *KIT* genotype (coded on the binary scale described above) into the logistic regression model.

The results of the statistical analysis were considered significant at *P*<0.05, whereas *P*⩽0.1 values were regarded as indicative of possible trends. As no Bonferroni-like adjustment for multiple testing was applied during this exploratory analysis, *P*-value nearing 0.05 has, however, to be considered cautiously. Proportional odds ratios related to free drug exposure were derived from the coefficients of the ordered logistic regression model (Stata Corp, 2003). The log_2_ of PK parameters (AUC and CL) and Dose was used for this calculation to obtain odds ratios corresponding to the effect of doubling the values.

## RESULTS

The 280 imatinib plasma concentration values considered ranged between 67 and 11 221 *μ*g l^−1^. The assessment of AGP plasma concentration in 51 patients (corresponding to 238 samples) provided results ranging from 0.4 to 3.2 g l^−1^. Among the 38 GIST patients, tumour *KIT* genotypes of 20 patients were available (corresponding to 111 different plasma samples). Various mutations were detected on the *KIT* gene: deletions, point mutations or mixed mutations in exon 11 (code=0; *n*=13), or alternately insertion in exon 9 (AY 502–503 duplication) or wt profile, that is no mutation (code=1; *n*=7). The patient demographics are presented in Table 1.

It is noteworthy that the type of pathology alone was in fact sufficient to predict the response (CML patients had globally better response scores than GIST patients, *P*<0.001). The results presented below refer to the per-sample analysis. Per-patient analyses gave similar trends, although without reaching significance.

### Concentration–effect exploration in CML patients

The pharmacodynamic exploration with total exposure revealed an inverse relationship between Dose, as well as AUC, and therapeutic response (*P*=0.073 for Dose and *P*=0.012 for AUC), with non-responding patients receiving higher doses than good responders. Similarly, a better response was associated with higher CL (*P*=0.023). A similar analysis carried out on toxicity scores showed that Dose and AUC were in turn positively correlated with the amount of side effects, although not significantly (*P*=0.062 for Dose and *P*=0.27 for AUC), whereas this was not the case for CL.

**Figure 1:**
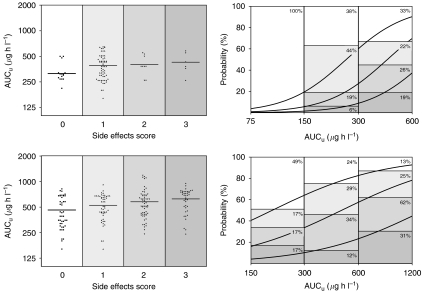
Relationship between free drug exposure (AUC_u_) and toxicity in CML (upper part) and GIST patients (lower part). Left panel: scatter plot of AUC_u_ according to side effects score (0=no side effects, 1=1 side effect, 2=2 side effects and 3=3 or more side effects). Right panel: probability of side effects according to the per-sample PK–PD analyses. The histograms represent the percentages observed for the three types of response at three typical AUC_u_ range values (side effects score: white box=0; light grey box=1; grey box=2; dark grey box=3). The curves, modelled by a four-level ordered logistic regression, show the probability of side effects according to AUC_u_.

Using free drug exposure estimates (derived from the AGP model previously mentioned) appeared to reverse the relationship between free AUC (AUC_u_) and response, although not significantly (*P*=0.48). Furthermore, free clearance (CL_u_) negatively correlated with the response (*P*=0.024). Concerning the tolerability to the drug, AUC_u_ remained positively correlated with the amount of side effects (*P*=0.013). The scatter plot of the upper part of Figure 1 depicts this relationship (left panel) as well as the ordered logistic regression curves (right panel). In the same analysis, CL_u_ also decreased with toxicity scores, although not significantly (*P*=0.33). The main results of this analysis in CML patients are presented in Table 2.

### Concentration–effect exploration in GIST patients, incorporating *KIT* genotype

A similar PK–PD analysis incorporating total drug levels in the GIST population again showed some inverse relationship between Dose, AUC or CL and therapeutic response (yet not reaching significance for Dose and CL). This logistic regression analysis also showed that the response tended to be affected by the mutation profile (*P*=0.071), with patients presenting a resistance-related profile (i.e. *KIT* exon 9 mutation or wt *KIT*) showing a lower response rate. In the tolerability analysis, Dose and AUC appeared positively and significantly correlated with the amount of side effects (*P*<0.001 for Dose and AUC), whereas this was still not the case with CL.

Using free drug exposure estimates (derived from the AGP model) did not change the general relationship between AUC_u_ and response (*P*=0.63). Concerning the tolerability of the drug, AUC_u_ remained positively correlated with the amount of side effects (*P*<0.001 in per-sample analysis). Regarding CL_u_, lower values tended to be associated with lesser side effects, albeit not reaching significance (*P*=0.063).

Finally, incorporating the genotype profile in the analysis using free level parameters improved to a noticeable degree the relationships previously observed. AUC_u_ indeed correlated with response (*P*=0.026), whereas CL_u_ appeared inversely linked to response, with lower clearance predicting better outcome (*P*=0.002). Importantly, AUC_u_ and CL_u_ appeared better predictors of the response than the mutation profile itself (affecting the response, but never significantly in multivariate analyses). Concerning toxicity, AUC_u_ also appeared to be a better predictor than the mutation profile (*P*=0.014 in multivariate analysis).

**Figure 2:**
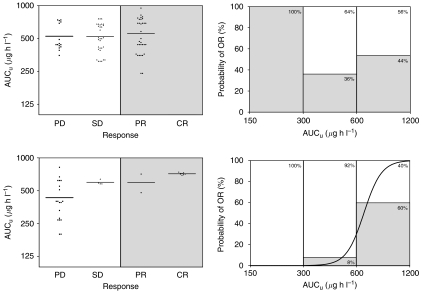
Relationship between free drug exposure (AUC_u_) and response in GIST patients. Upper part: exon 11 *KIT* genotype; lower part: exon 9 or wt *KIT* genotype. Left panel: scatter plot of AUC_u_ according to RECIST score; white box=PD+SD (score 0; *n*=23 for exon 9/wt, 46 for exon 11); grey box=OR, OR=CR+PR (score 1; *n*=10 for exon 9/wt, 32 for exon 11). Right panel: probability of response according to the per-sample PK–PD analysis for both main genotypes of GIST patients. The histograms represent the percentages observed for the two types of response at three typical AUC_u_ range values. The curve, modelled by a two-level ordered logistic regression, shows the probability of response according to AUC_u_.

Figure 2 depicts the results of the per-sample concentration–effect analysis with the associated logistic regression curves (probability of response *vs* AUC_u_). With exon 11, this curve could not be modelled (no significant differences in response according to AUC_u_). The histograms represent the percentage of the two types of response at three typical AUC_u_ range values. Table 2 also presents the main results related to this GIST population analysis.

## DISCUSSION

This clinical exploration reveals that three main confounders can obscure the PK–PD relationship of imatinib: dose selection, protein binding and genetic heterogeneity of the target tumour. Taking into account those three factors allowed observing clearer concentration–response effects.

Several studies had actually suggested that the administration of higher doses than the typical 400 mg daily regimen could improve the response in some patient subsets. A better response was indeed observed in accelerated and blast phases of CML with 600 mg daily (Talpaz *et al*, 2002), and a 800 mg daily regimen allowed a longer progression-free survival in GIST patients (Verweij *et al*, 2004), whereas this was not the case with 600 mg (Blanke *et al*, 2008).

The inverse relationship initially observed in our PK–PD analysis (for both CML and GIST patients) between Dose/AUC and therapeutic response could be considered paradoxical. However, as our study was purely observational, we were in the presence of good responders selected to receive low doses and bad responders high doses, but without apparent advantage. In GIST patients, Dose was indeed highly correlated with AUC and CL, confirming the presumption of such a bias. Conversely, in the CML sub-population, the lower the clearance of the unbound drug, the better was the response, suggesting that CL_u_ was a better predictor of effect than AUC/AUC_u_. Most CML patients were apparently exposed to sufficient drug amounts to achieve a haematological response (i.e. ceiling of the concentration–effect curve), making them partly obscure the PK–PD relationship. It has indeed been reported that imatinib doses of 350 mg (corresponding to a trough plasma concentration, TPC of 570 *μ*g l^−1^) already ensure an optimal haematological response in CML (Peng *et al*, 2004). Such an amount could, however, not be sufficient for a cytogenetic or molecular response, which appears to require TPC as high as 1000 *μ*g l^−1^ (Picard *et al*, 2007; Larson *et al*, 2008).

Moreover, the design of our study wherein AUC derived from sparse measurements were used as an index of exposure may have prevented us from observing similar results as in the IRIS study (steady-state imatinib TPC at initiation of therapy in patients on 400 mg QD predicts long-term complete cytogenetic and major molecular responses) (Larson *et al*, 2008). As the PK–PD relationship for a targeted agent such as imatinib may be confounded by genotypic heterogeneity of intracellular pharmacological targets (BCR-ABL and c-KIT, respectively), the mutational status of *BCR-ABL* was also assessed in our CML population by DNA sequencing. However, no point mutations known to confer resistance were observed (data not shown).

Conversely, focusing on GISTs allowed us to uncover a relationship between free drug exposure and response when integrating the target mutation profile (with higher drug exposure predicting better response, and being a superior predictor than the mutation status). Of importance, the inclusion of SD in the OR score did not significantly affect the correlations observed. Imatinib-free plasma levels thus appeared a better predictor of drug effect than total levels. This is in line with previous data showing that the total plasma concentration of imatinib is a poor marker of imatinib clinical effect (Delbaldo *et al*, 2006). Very recently, however, Demetri presented data showing that imatinib total TPC could correlate with response (expressed also as OR=CR+PR) in a larger GIST population, and this more significantly than AUC (Demetri *et al*, 2008).

On the basis of our data, Figure 2 suggests that patients with tumours harbouring a ‘sensitive’ c-KIT genotype (*KIT* exon 11 mutations) are exposed to concentrations that are already near the top of the concentration–response curve (as was probably the case in our CML patients; see above). On the other hand, patients with a ‘resistant’ genotype (exon 9 mutations or wt *KIT*) are probably lying in the steep part of the curve, where a definite concentration–response relationship can be observed. Such patients could probably draw the most benefit from a thorough adjustment of their imatinib exposure. It has indeed been demonstrated that patients harbouring an exon 9 mutation benefit the most from a 800 mg daily regimen (Debiec-Rychter *et al*, 2006). When taking into account the mutation profile in our analysis, lower CL_u_ also proved to predict better responses in both groups. Again, the poor correlation between concentration and response observed without considering the mutation profile suggests that this relationship could be obscured by a Dose selection effect.

Our study has thus been able to demonstrate for the first time a clear relationship between exposure to the unbound drug and clinical efficacy of imatinib in GIST patients. It provides a clinically relevant PK–PD model using logistic regression with formal assessment of *in vivo* concentration–effect curves, instead of a mere comparison of PK parameters (e.g. TPC) between responders and non-responders. Additionally, our PK–PD exploration formally established that the occurrence of side effects is more frequent at higher imatinib exposure levels (Figure 1). Together with previous data (Delbaldo *et al*, 2006), this indicates that monitoring imatinib plasma levels may help to identify patients with unnecessarily high levels at risk of developing toxicity. In the literature, several cases have indeed been reported where imatinib treatment had to be discontinued because of the occurrence of serious adverse events (Brouard and Saurat, 2001; Elliott *et al*, 2002; Gambillara *et al*, 2005; Blasdel *et al*, 2007). In some cases, plasma drug measurement and dose adjustment were considered (Blasdel *et al*, 2007; Gambillara *et al*, 2005). Concerning our data, it is worth noting, however, that a severity scale should have been used (typically NCI-CTC). As mentioned above, it was not available at the time of our study. The incidence scale used instead has been applied elsewhere (Schuell *et al*, 2005), but it has to be considered cautiously and may prevent formal comparison with other studies. It, however, allowed a general delineation of concentration–toxicity relationships.

Our exploratory study (performed on a small patient set), associated to data already published for CML (Picard *et al*, 2007; Larson *et al*, 2008) and for GIST (Demetri *et al*, 2008), should thus stimulate further confirmation in larger populations of the relationship between imatinib exposure, suitably free plasma level, and its efficacy and toxicity. A prospective study to validate a therapeutic drug monitoring (TDM) approach is indeed being initiated in France (Picard *et al*, 2007). Such paradigms will potentially apply to other new targeted anticancer drugs under development or already approved by registration authorities. For instance, it has recently been shown in an animal model that tumoural phospho-BCR-ABL inhibition is directly correlated with plasma levels of dasatinib, a novel BCR-ABL inhibitor (Luo *et al*, 2006). For imatinib, the additional monitoring of the active N-demethylated metabolite may also be considered (Delbaldo *et al*, 2006). Our data also suggest that patient stratification by genotype will be important for future investigation. As recently stated, molecular subclassification is becoming an important element for providing personalised care to oncologic patients (Heinrich and Corless, 2006).

In conclusion, the various PK–PD relationships progressively uncovered, together with some case reports on the benefit of such an approach in imatinib treated patients (Blasdel *et al*, 2007), provide arguments to evaluate further the potential benefit of a TDM programme in well-controlled clinical trials. As recently declared by Brian Druker (quoted in Tuma, 2007), targeted anticancer drugs treatment may follow the HIV model, notably by combination therapy (see also Stebbing and Bower, 2003). In HIV patients, TDM is increasingly recommended (e.g. for drug interactions, in case of toxicity and for drug exposure assessment) in association with the viral genotype profile. Therefore, in oncology, an approach that integrates clinical PKs and patient/tumour pharmacogenetics may well contribute to optimise the therapeutic use of new drugs, such as signal transduction inhibitors, in patients.

## Figures and Tables

**Table 1:** Patient demographics of the 58 patients evaluated in this concentration–effect analysis (providing 280 plasma samples)

	**Patients**		**Samples**	
**Characteristic**	**CML**	**GIST**	**CML**	**GIST**
*Pathology diagnosis (no.)*				
GIST		38		227
CML	20		53	
				
*Gender (no.)*				
Men	9	24	23	138
Women	11	14	30	89
				
*Age (years)*				
Median			48	57
Range			27–71	20–79
				
*Imatinib daily dose (mg)*				
Median			400	600
Range			150–800	200–800
				
*AGP plasma levels (g per l)*				
Median			0.7	0.9
Range			0.4–3.0	0.4–3.2
				
*KIT genotype (no.)*				
Exon 11 mutation		13		78
Exon 9 mutation or wt *KIT*		7		50
Not available		18		99
				
*Side effects incidence (no.)*				
0			19	52
1			15	62
2			10	68
3 or more			9	45
				
*Haematological Response score (no.)*				
No response (NHR)			7	
Partial response (PHR)			20	
Complete response (CHR)			26	
				
*RECIST response score (no.)*				
Progressive disease (PD)				58
Stable disease (SD)				72
Partial response (PR)				87
Complete response (CR)				10
				
*Dichotomous response (no.)*				
Progressive or stable disease (SD+PD)				130
Overall responses (OR=PR+CR)				97

**Table 2:** Results of the per-sample multivariate logistic regression analysis related to total and free drug exposure

	**Total exposure**							
	**All CML^a^**		**All GIST**		**Exon 11 mutation GIST**		**Exon 9 mutation or wt GIST**	
	**53**		**227**		**86**		**36**	
***n***	**OR**	***P*-value**	**OR**	***P*-value**	**OR**	***P*-value**	**OR**	***P*-value**
*Response vs*								
Dose	0.3 (±0.2)	0.073	0.7 (±0.2)	0.206	0.6 (±0.2)	0.306	→ ∞^b^	0.008
mut			0.8 (±0.2)	0.645	1.4 (±0.6)	0.398	0.9 (±0.6)	0.821
AUC	0.5 (±0.1)	0.012	0.5 (±0.1)	0.012				
AUC			1.2 (±0.4)	0.541				
+ mut			0.8 (±0.3)	0.661	0.5 (±0.2)	0.082	8.9 (±8.0)	0.015
CL	2.6 (±1.1)	0.023	1.5 (±0.4)	0.097				
CL			1.0 (±0.3)	0.976				
+ mut			0.8 (±0.3)	0.646				
								
*Toxicity vs*								
Dose	3.2 (±2.1)	0.062	2.8 (±0.7)	0.000	2.4 (±0.9)	0.027	3.3 (±2.5)	0.114
mut			1.9 (±0.8)	0.071	1.2 (±0.4)	0.544	0.5 (±0.3)	0.224
AUC	1.4 (±0.4)	0.265	2.2 (±0.4)	0.000				
AUC			1.0 (±0.3)	0.906				
+ mut			1.9 (±0.7)	0.071	0.5 (±0.3)	0.147	3.2 (±1.7)	0.032
CL	0.9 (±0.4)	0.816	0.9 (±0.2)	0.708				
CL			2.0 (±0.6)	0.017				
+ mut			1.7 (±0.6)	0.132				
								
	**Free exposure**							
	**44**		**193**		**78**		**33**	
***n***	**OR**	***P*-value**	**OR**	***P*-value**	**OR**	***P*-value**	**OR**	***P*-value**
*Response vs*								
Dose	0.4 (±0.3)	0.242	0.8 (±0.3)	0.630	0.7 (±0.3)	0.321	→ ∞^b^	0.037^c^
mut			0.6 (±0.3)	0.289	1.3 (±0.6)	0.621	→ ∞^b^	0.029
AUC_u_	1.6 (±1.0)	0.481	0.9 (±0.2)	0.548				
AUC_u_			2.6 (±1.1)	0.026				
+ mut			0.6 (±0.2)	0.299	0.1 (±0.1)	0.007	0.1 (±0.1)	0.104
CL_u_	0.8 (±0.9)	0.024	1.2 (±0.5)	0.750				
CL_u_			0.1 (0.1)	0.002				
+ mut			1.1 (0.6)	0.807				
								
*Toxicity vs*								
Dose	7.2 (±6.2)	0.022	2.4 (±0.7)	0.001	2.1 (±0.8)	0.064	4.9 (±4.0)	0.051
mut			1.7 (±0.6)	0.167	2.1 (±1.0)	0.118	3.7 (±2.3)	0.035
AUC_u_	6.1 (±4.5)	0.013	2.7 (±0.6)	0.000				
AUC_u_			2.4 (±0.9)	0.014				
+ mut			1.7 (±0.6)	0.148	2.4 (±1.8)	0.240	0.2 (±0.2)	0.154
CL_u_	0.4 (±0.4)	0.330	0.5 (±0.2)	0.063				
CL_u_			1.3 (±0.8)	0.726				
+ mut			1.6 (±0.6)	0.241				

OR=odds ratios associated with doubling value of the predictor (AUC/AUC_u_, CL/CL_u_, Dose) or with mutation profile (mut).

PK parameters expressed as log_2_ values; response on a 3-point scale for CML and 2-point scale for GIST (OR *vs* SD+PD), tolerability on a 4-point scale, and dichotomous mutation profile. The odds ratios (± s.e.) represent the effect on efficacy and toxicity score of a doubling of the PK parameter (AUC/AUC_u_ or CL/CL_u_) or the Dose.

a No BCR-ABL mutation detected in the CML population.

b Groups entirely distinct.

c Approximated value only, due to a lack of sufficient different samples.
